# SUCCESSFUL TREATMENT OF SEVERELY CHOLESTATIC ACUTE HEPATITIS B WITH ORAL CORTICOSTEROID

**DOI:** 10.1590/0102-672020190001e1511

**Published:** 2020-08-24

**Authors:** Bipadabhanjan MALLICK, Preetam NATH, Dibya L PRAHARAJ, Sarat C. PANIGRAHI

**Affiliations:** 1 Department of Gastroenterology and Hepatology, Kalinga Institute of Medical Science, Bhubaneswar, India

**Keywords:** Hepatitis B, Cholestasis, Corticosteroid, Hepatite B, Colestase, Corticosteroide

## INTRODUCTION

Acute hepatitis B (HBV) infection is asymptomatic, subclinical illness in approximately two thirds of cases and diagnosis is made only through serologic testing^7^. Clinical evidence of hepatitis; jaundice and occasionally acute liver failure develops in rest one third of patients with acute HBV infection^7^. Cholestatic hepatitis is one of very rare manifestations of acute HBV infection, but when develop it leads to prolonged hospital stay and increased medical expenses^5^. Rapid improvement of the clinical symptoms and signs have been reported in patients with cholestatic hepatitis A (HAV) after use of corticosteroid^6^. We here report a case of cholestatic hepatitis due to acute HBV infection and rapid improvement of symptoms with oral prednisolone. 

## CASE REPORT

A 56-year old female patient presented with complaints of progressively increasing jaundice and intense pruritus that disturbed sleep for 12 weeks duration. She had been evaluated at a local hospital and diagnosed to have acute viral hepatitis due to hepatitis B virus infection (HBV) on the basis of raised liver enzymes and serological markers (Table 1). 

**TABLE 1:** Serological markers and liver function test at initial presentation to a health care system

Parameters	Result
HBsAg	Reactive
IgM anti HBc antibody	Reactive
HBeAg	Reactive
Anti HBe antibody	Non-reactive
HBV DNA	1 x 105 IU/ml
Bilirubin (Total/Direct)	2.3 mg/dl/ 1.2 mg/dl
AST/ALT	1408 U/L, 1670 U/L
GGT/ALP	59 U/L, 122 U/L

There was no history of drug ingestion, blood transfusions, or similar episodes in past. She had a soft and tender liver, palpable 2 cm below the right costal margin (liver span: 9 cm). At presentation to our centre, the serum bilirubin level was 29.9 mg/dl (direct fraction 24.5 mg/dl) with serum aspartate transaminase (AST) of 267 U/L, alanine transaminase (ALT) of 110 U/l, alkaline *phosphatase* (ALP) of 212 U/l and gamma-glutamyl transferase (*GGT*) of 78 U/l. She had no biochemical evidence of hepatic biosynthetic defect with serum albumin levels of 3.7 gm/dl and INR of 1.1. Ultrasonography showed hypoechoic liver and normal intra and extrahepatic bile ducts. She was started with tenofovir*disoproxil*fumarate 300 mg and for cholestasis she was treated with *ursodeoxycholic acid* (UDCA, 450 mg, twice daily), cholestyramine (4 g, twice daily) and rifampicin (150 mg, once daily). Investigations were done to detect other causes of prolonged intra-hepatic cholestasis: tests for detection of antibodies against hepatitis A, hepatitis E, and nuclear, smooth muscle, mitochondrial, and liver-kidney antigens were negative. The serum ceruloplasmin was 40 mg/dl and thyroid function was normal. Re-evaluated at eight weeks after starting the treatments with no improvement in pruritus, laboratory work-up showed: total bilirubin: 26.7 mg/dl, direct bilirubin: 22.9, AST: 243 U/l, ALT: 62 U/l, GGT: 64 U/L, ALP:200 U/l, mg/dl, INR:1.2, serum albumin 2.9 gm/dl and normal magnetic resonance cholangiopancreatography (MRCP).

**FIGURE 1:**
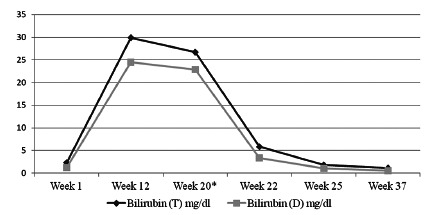
Bilirubin changes in the index patient

Oral prednisolone was then started with 40 mg/day for prolonged symptomatic cholestasis in the absence of any other chronic cholestatic liver disease. Within a week, there was a dramatic reduction in pruritus, jaundice started regressing. The prednisolone dose was tapered to 20 mg/day after two weeks when the serum bilirubin levels decreased to 5.8 mg/dl, after which the dose was tapered gradually and steroids were withdrawn after total five weeks and at end of steroid therapy her bilirubin level was 1.8 mg/dl (Figure 1). At follow-up, 12 weeks after the steroid therapy, the patient continues to be asymptomatic, HBsAg loss, HBV DNA 34 IU/ml; has not shown any clinical or biochemical evidence of relapse.

* Prednisolone started

## DISCUSSION

Hepatitis B (HBV) infection is endemic in Asia and the Pacific islands, Africa, Southern Europe and Latin America^7^. On the basis of immune interactions between virus and host, HBV infection can have diverse clinical manifestations, including acute hepatitis, chronic hepatitis, liver cirrhosis, and hepatocellular carcinoma^7^. However, cholestatic hepatitis due to HBV infection were reported very rarely in literature^5^. Our patient represents a typical case of acute HBV infection acquired at the age of 56 years and expected to resolve spontaneously. Her course got complicated by cholestatic hepatitis and we attributed it to HBV infection after ruling out all possible causes of intrahepatic and extrahepatic causes. 

Cholestasis accompanied by HBV infection is mainly seen in immunosupressed patients especially post transplant patients known as *fibrosing cholestatic hepatitis*(FCH) and it is due to direct effect of virus^8^. The exact cause of cholestasis in HBV infection is not known. Approximately 25% of hepatitis B biopsy samples show portal reversible lymphoid aggregates or follicles, and less than 10% reveal bile duct damage^3^ ^,^ ^4^. Hepatitis B surface and core antigens have also been demonstrated in cholangiocytes in a small minority of cases^2^. Necro-inflammatory changes of zone 1 hepatocytes in HBV infection also leads to ductular metaplasia^1^. Probably these are the reason for cholestasis in HBV infection and it is not clear whether it is direct effect of virus or immune interaction. Non-response to antiviral agents and rapid response to corticosteroid in index patients indicate towards immune interaction etiology. 

Our patient remained symptomatic despite use of UDCA, cholestyramine and rifampicin and needed some intervention for relief of symptoms. Corticosteroid had been successfully used for HAV associated prolonged cholestasis, so we planned to treat cholestasis with oral prednisolone as our patient had already been started on tenofovir *disoproxil*fumarate for high bilirubin level. Prednisolone therapy was administered for five weeks to our patient and was not associated with any clinical or biochemical deterioration during or after that period. 
